# Listening to children’s and parents’ voices: using patient reported outcomes to empower patients with orphan diseases and their parents

**DOI:** 10.1186/1750-1172-7-S2-A30

**Published:** 2012-11-22

**Authors:** Linda Abetz-Webb

**Affiliations:** 1 Rob Arbuckle, Adelphi Values, Bollington, Cheshire, UK

## Background

Systematic evaluation of outcomes is essential for clinical trial research, yet outcomes often neglect the voice of the child and parent, particularly within paediatric orphan diseases.

While guidance for the development and validation of Patient Reported Outcomes (PROs) and Observer Reported Outcomes (ObsRO) measures are available from EMA and FDA , little attention has focused on paediatric and orphan disease PRO/ObsRO development methods[1,2].

## Methods

We summarize considerations for the development, validation and use of paediatric measures and provide examples of their successful use.

## Results

Table 1 provides the recommended steps for PRO/ObsRO development, as well as the challenges and potential solutions in PRO/ObsRO development in paediatrics and orphan diseases. When developing paediatric measures, it is critical to use developmentally appropriate language and techniques to ensure measures have content validity and will be reliable and valid.

**Table 1:** Challenges and potential solutions for PRO/ObsRO development and implementation in paediatrics and orphan indications

Steps to PRO/ObsRO development	Challenges	Solutions
Literature Review/Desk Research	• Literature often limited • Broad age ranges covered	• Consider grey literature (blogs, dissertations) • Conduct interviews with expert clinicians, nurses and patient advocacy groups

Concept Elicitation	• Limitations in memory, cognitive ability, language by age/condition • Children can be shy • Rarity of condition makes recruitment/saturation hard to achieve • Parents unable to report some symptoms/domains not known to them	• Carefully guided interview guides and well trained interviewers • Creative interview techniques, toys and drawings • Collapse age groups where appropriate • Must achieve saturation within each narrow age range – can this be relaxed for orphan indications? Get FDA feedback early • Consider other respondents (teachers, nanny etc)

Selection/ development of a measure	• Few disease specific measures exist in paediatrics and orphan diseases • Existing instruments don’t meet FDA/EMA guidance • Who is the best respondent? • How should you administer the questionnaire? • How should the questionnaire be worded? • Child can’t remember without a concrete event to recall to • Parent items must be observable….but they may not be with the child all day	• Think about PRO selection early • Talk to patient advocacy groups • Engage FDA early • Consider EPRO vs pen/paper vs IVRS in context of condition and age of child • Questions/responses should be clear and simply worded • Short recall period required • Consider all types of respondents (parent, teacher, nurse, child, dr) • Consider ‘child told me’ questions

Cognitive debriefing/ content validity testing	• Hypothetical situations don’t work with children • Children give you answers they think you want to hear • Small sample sizes in rare conditions	• Allow child to complete diaries at home for a few days prior • Use carefully worded interview guides and well trained interviewers • Questioning should not be too repetitive nor lengthy • When analysing, check for consistency between behaviour and responses • Collapse age groups as appropriate

Psychometric validation	• Sample should be stratified by age group, but small samples in orphan indications	• Consider validating as part of trial and/or include data from cognitive debriefing (move forward at risk) • Consider collapsing across age groups • Consult regulatory early • Utilize psychometrics done in other diseases if adapting a measure

Concept elicitation (using qualitative research) and psychometric validation require samples sizes within narrow age bands (0-2, 3-5, 6-8, 9-11, 12-14, 15-17), but also need to consider rates of development within the context of the condition being studied. From 0-5 years, parents are asked about child behaviours they observe that are indicative of symptoms or impact. For 6-11 years, the child and parent should be asked though simple questions, with images attached to responses, should be used for the child. For 12-17 years, more adult language (without jargon) can be used. Pooling data across ages can only be considered if different age versions are shown to be conceptually equivalent.

PROs have been used to aid decision making for regulatory approval (as in the case of Icatibant for the treatment of Heriditary Angioedema)[3-5] and reimbursement (as in the case of Exjade for the treatment of iron overload for B-thallaesemia and sickle cell disease)[6,7].

## Conclusions

Strong paediatric PRO/ObsRO research is needed to ensure ‘fit for purpose’ outcomes are used in paediatric trials to collect robust evidence regarding the safety and efficacy of drugs for children who have orphan diseases. When successfully implemented, all stakeholders benefit: regulators, payers, doctors, parents and most importantly, the children themselves.
